# Microbial DNA extraction method for avian feces and preen oil from diverse species

**DOI:** 10.1002/ece3.70220

**Published:** 2024-09-02

**Authors:** Austin C. Russell, Margaret A. Kenna, Alex Van Huynh, Amber M. Rice

**Affiliations:** ^1^ Department of Biological Sciences Lehigh University Bethlehem Pennsylvania USA; ^2^ Department of Biology DeSales University Center Valley Pennsylvania USA

**Keywords:** Aves, bacterial symbionts, feces, microbiome, uropygial gland

## Abstract

As DNA sequencing technology continues to rapidly improve, studies investigating the microbial communities of host organisms (i.e., microbiota) are becoming not only more popular but also more financially accessible. Across many taxa, microbiomes can have important impacts on organismal health and fitness. To evaluate the microbial community composition of a particular microbiome, microbial DNA must be successfully extracted. Fecal samples are often easy to collect and are a good source of gut microbial DNA. Additionally, interest in the avian preen gland microbiome is rapidly growing, due to the importance of preen oil for many aspects of avian life. Microbial DNA extractions from avian fecal and preen oil samples present multiple challenges, however. Here, we describe a modified PrepMan Ultra Sample Preparation Reagent microbial DNA extraction method that is less expensive than other commonly used methodologies and is highly effective for both fecal and preen oil samples collected from a broad range of avian species. We expect our method will facilitate microbial DNA extractions from multiple avian microbiome reservoirs, which have previously proved difficult and expensive. Our method therefore increases the feasibility of future studies of avian host microbiomes.

## Graphical abstract

Across many taxa, microbiomes can have important impacts on organismal health and fitness, but in order to study microbiomes in birds, microbial DNA must be successfully extracted from challenging sample types. Here, we present a microbial DNA extraction method that is less expensive than other commonly used methodologies and is highly effective for both fecal and preen oil samples collected from a broad range of avian species.

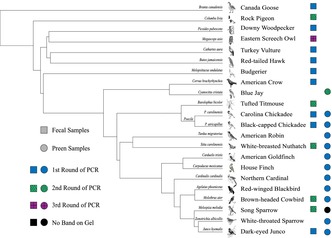

## 1 INTRODUCTION

Animal microbiomes have received increased attention in recent years (Abughazaleh et al., 2021; Bahrndorff et al., 2016; Barko et al., 2018; Bodawatta, Hird, et al., 2021; Esser et al., 2019; Gilroy, 2021; Hasan & Yang, 2019; Kau et al., 2011; Kogut & Fernandez‐Miyakawa, 2022; Kohl, 2012; Loftus et al., 2021). Further, the natural microbial communities that live on a host organism are known to positively impact health, pathogen resistance, and digestive capabilities and aid in other natural bodily functions (Bodawatta, Hird, et al., 2021; Bodawatta, Koane, et al., 2021; Broom & Kogut, 2018; Davidson et al., 2020; Drobniak et al., 2022; Trevelline & Kohl, 2022). Environment, dietary niche, and the health of a host all influence the diversity and abundance of different bacterial species present in a host organism (Bodawatta, Hird, et al., 2021). Avian species are globally widespread and play many important roles in their ecosystems (Şekercioğlu et al., 2004; Wenny et al., 2011; Whelan et al., 2008). Understanding the factors that contribute to health and fitness in birds has become especially important in recent decades due to the rapid decline in bird populations worldwide (Rosenberg et al., 2019; Şekercioğlu et al., 2004). Thus, an understanding of the different microbiomes present within and across avian species provides valuable insights about avian health, ecology, evolution, and conservation. A necessary component of characterizing such microbiomes is the accurate and effective identification of microorganisms from specific reservoirs.

Although host species possess many reservoirs for microbial species, one of the most important is the gut (Bahrndorff et al., 2016; Barko et al., 2018). Avian gut microbiomes and their relationship to the behavior and fitness of the host have been of great interest over the past two decades (Bodawatta, Freiberga, et al., 2021; Davidson et al., 2018, 2020; Drobniak et al., 2022; Gilroy, 2021; Hu et al., 2017). Specifically, the microbiota that exist in the gut of a bird have been linked to behavior, cognition, metabolic function, and digestion (Bodawatta, Hird, et al., 2021; Davidson et al., 2018, 2020; Drobniak et al., 2022; Goncerzewicz et al., 2022; Grond et al., 2018; Hird et al., 2015; Juan et al., 2020; Trevelline & Kohl, 2022). To study the gut microbiome, most scientists extract bacterial DNA from fecal samples. Although fecal samples do not perfectly represent the gut microbiome without bias, most microbiomes have been characterized using bacterial DNA extracted from fecal samples because it allows the characterization of the bacteria present in the stomach and along the digestive tract as well (Ingala et al., 2018; Videvall et al., 2018).

In addition to gut microbiota, preen gland microbiota are important for birds (Bodawatta, Hird, et al., 2021; Bodawatta, Koane, et al., 2021; Noguera et al., 2018; Palinauskas et al., 2022; Parois et al., 2017; Petrullo et al., 2022; Stevens & Hume, 1998; Uebanso et al., 2020). The avian preen gland (or uropygial gland) is located at the base of the tail and produces oils that help birds clean themselves, protect feather health, and potentially aid in fighting against pathogenic bacteria (Huynh & Rice, 2021; Jacob et al., 2014; Møller & Laursen, 2019; Whittaker & Theis, 2016). Birds stimulate this gland with their beaks and then spread the resulting oil throughout their feathers in a behavior called preening (Whittaker et al., 2019). Besides feather health, chemicals from preen oils vary between species (Soini et al., 2013) and can mediate communication between individuals in many contexts (Moreno‐Rueda, 2017), including species recognition (Huynh & Rice, 2019; Zhang et al., 2010) and mate choice (Whittaker et al., 2011). Additionally, preen oil chemistry has been shown to vary with seasons (Whittaker et al., 2010), aggression levels (Whittaker et al., 2018), and reproductive success (Whittaker et al., 2013). Interestingly, recent studies have shown that preen oil contains bacteria (Bodawatta, Schierbech, et al., 2020; Grieves, Gloor, Bernards, et al., 2021; Grieves, Gloor, Kelly, et al., 2021; Rodríguez‐Ruano et al., 2015; Soler et al., 2008; Videvall et al., 2021; West, Digby, et al., 2022), which play a role in the synthesis of the chemical compounds found in the oil (Martín‐Vivaldi et al., 2009; Whittaker et al., 2019). Thus, both gut and uropygial gland microbiota play important roles in avian life.

To characterize the microbiomes present within a species, microbial DNA must be extracted from samples collected from different reservoirs. To facilitate comparisons of the microbiome communities present in different reservoirs, the field would benefit from a microbial DNA extraction method that is effective for different sample types. However, within avian hosts, multiple sample types present particular difficulties. Specifically, microbial DNA extractions from both avian fecal material and preen oil present several challenges. Although feces contain abundant bacteria, avian and reptilian organisms combine their feces with urine, which contains minimal bacteria and therefore lowers the overall concentrations of the bacteria present in fecal samples (Khan et al., 1991; Mahony et al., 1998; Munch et al., 2019). Further, urine contains PCR inhibitors, such as uric acid, which lower the efficiency of DNA polymerase (Munch et al., 2019) and impede bacterial DNA detection (Alcaraz et al., 2016; Eriksson et al., 2017; Gerasimidis et al., 2016; Khan et al., 1991; Kirschner et al., 2004; Mahony et al., 1998; Munch et al., 2019; Videvall et al., 2018). Additionally, preen oil displays low bacterial abundance (Soini et al., 2013) and exhibits antimicrobial properties (Martín‐Vivaldi et al., 2009), leading to an expectation of even lower bacterial yields from preen gland versus avian fecal samples (Alcaraz et al., 2016). The preen oil microbiome has only recently begun to receive attention in the literature and there are no standardized methodologies for preen gland microbial DNA extraction (Bodawatta, Schierbech, et al., 2020; Grieves, Gloor, Bernards, et al., 2021; Grieves, Gloor, Kelly, et al., 2021; Rodríguez‐Ruano et al., 2015; Videvall et al., 2021).

Most studies on microbiomes have used commercial DNA extraction kits that are used on other sources of microbial DNA such as human feces or soil (Alcaraz et al., 2016; Bodawatta, Puzejova, et al., 2020; Eriksson et al., 2017; Gerasimidis et al., 2016; Kirschner et al., 2004; Videvall et al., 2018). For avian fecal matter however, DNA extractions from commercial kits often do not produce visible bands on agarose gels, complicating verification of extraction success. For example, Eriksson et al. (2017) compared the performance of six different commercial DNA extraction kits using mallard duck (*Anas platyrhynchos*) feces and few bands were visible on gel images. Additionally, the commercially available fecal extraction kits (Eriksson et al., 2017; McOrist et al., 2002) can be quite expensive (Table 1).

**TABLE 1:** Cost comparison of three commercial fecal DNA extraction kits with our extraction method.

Extraction method	Source of reagents/kit	Total cost	No. of extractions	Cost per extraction (US dollars)
MP Biomedicals™ FastDNA™‐96 Fecal DNA Extraction Kit	Fisher Scientific	$1426	192	$7.43
QIAamp PowerFecal Pro DNA Kit	Qiagen	$451	50	$9.02
Quick‐DNA Fecal/Soil Microbe Miniprep Kit	Zymo‐Research	$249	50	$4.98
Russell et al. extraction method	Thermo Fisher Scientific and Fisher Scientific	$320	100	$3.20

Thus, there is a need for more effective, efficient, and inexpensive methodologies for extracting microbial DNA from multiple reservoirs. Here, we present a new method that effectively and consistently extracts bacterial DNA from both fecal and preen oil samples collected across a wide range of avian species.

## 2 MATERIALS AND METHODS

### 2.1 Fecal and preen oil sampling

We obtained fecal samples from a total of 25 individual birds across 15 species (14 genera, 12 families, and 7 orders; Table 2). To capture all but one of the Passerines used in this study (excluding the American crow; see Table 2), we used mist nets at feeders in Northampton and Lehigh Counties in Pennsylvania, USA (U.S.G.S. Federal Banding Permits 23810 to AMR and 24256 to AVH; Pennsylvania Banding Permits 103 to AMR and 49864 to AVH). We briefly held each bird individually in a cage containing a clean cage liner until they defecated, which usually took no longer than several minutes. We then immediately collected the feces using tweezers. We also collected fecal samples from two budgerigars (*Melopsittacus undulatus*) housed in a local pet store (PetSmart in Bethlehem, Pennsylvania, USA). Tweezers were cleaned by wiping with ethanol before and after each use. We transferred the feces into a 1.5 mL microcentrifuge tube containing 100% ethanol and stored the samples in a −80°C freezer until microbial DNA extraction. Although ethanol kills the living bacteria, it preserves all the DNA present in the sample so that any bacteria present can be detected even after freezer storage (Srinivasan et al., 2015).

**TABLE 2:** Avian taxa from which we obtained fecal samples.

Class	Order	Family	Genus	Species	Common name	Feeding guild	No. of samples
Aves	Anseriformes	Anatidae	Branta	*Branta canadensis*	Canada goose	Granivore, Graminivore	2
Aves	Columbiformes	Columbidae	Columba	*Columba livia*	Rock pigeon	Granivore	2
Aves	Accipitriformes	Cathartidae	Cathartes	*Cathartes aura*	Turkey vulture	Scavenger	1
Aves	Accipitriformes	Accipitridae	Buteo	*Buteo jamaicensis*	Red‐tailed hawk	Carnivore	1
Aves	Piciformes	Picidae	Dryobates	*Picoides pubescens*	Downy woodpecker	Insectivore	1
Aves	Strigiformes	Strigidae	Megascops	*Megascops asio*	Eastern screech owl	Carnivore	1
Aves	Psittaciformes	Psittaculidae	Psittacula	*Melopsittacus undulatus*	Budgerigar	Frugivore, Granivore	2
Aves	Passeriformes	Corvidae	Corvus	*Corvus brachyrhynchos*	American crow	Omnivore	1
Aves	Passeriformes	Paridae	Baeolophus	*Baeolophus bicolor*	Tufted titmouse	Insectivore	2
Aves	Passeriformes	Paridae	Poecile	*Poecile carolinensis*	Carolina chickadee	Insectivore, Frugivore	4
Aves	Passeriformes	Paridae	Poecile	*Poecile atricapillus*	Black‐capped chickadee	Insectivore, Frugivore	3
Aves	Passeriformes	Sittidae	Sitta	*Sitta carolinensis*	White‐breasted nuthatch	Insectivore	1
Aves	Passeriformes	Icteridae	Molothrus	*Molothrus ater*	Brown‐headed cowbird	Granivore	1
Aves	Passeriformes	Passerellidae	Junco	*Junco hyemalis*	Dark‐eyed junco	Granivore	2
Aves	Passeriformes	Passerellidae	Melospiza	*Melospiza melodia*	Song sparrow	Insectivore	1

Additionally, in cooperation with the Wildlands Conservancy Nature Preserve in Lehigh County, Pennsylvania, we obtained fecal samples from captive birds including the American crow (*Corvus brachyrhynchos*), plus several species spanning four additional orders (Table 2): red‐tailed hawk (*Buteo jamaicensis*), eastern screech owl (*Megascops asio*), turkey vulture (*Cathartes aura*), and rock pigeon (*Columba livia*). The Wildlands Conservancy samples were collected as the individuals were seen defecating in their enclosures. The collections were placed in fresh Ziploc® bags rather than in ethanol‐filled tubes and placed in a freezer prior to extraction. To collect from the Canada goose (*Branta canadensis*), we closely observed wild geese on the DeSales University campus. When we saw defecation, we used ethanol‐wiped tweezers to lift a small portion of the fresh fecal samples off the ground and placed them into individual tubes of ethanol. Because the Canada goose produces larger feces than the other species we sampled, we were careful to avoid any white portions of these fecal samples, with the goal of reducing the amount of uric acid in the samples.

**FIGURE 1:**
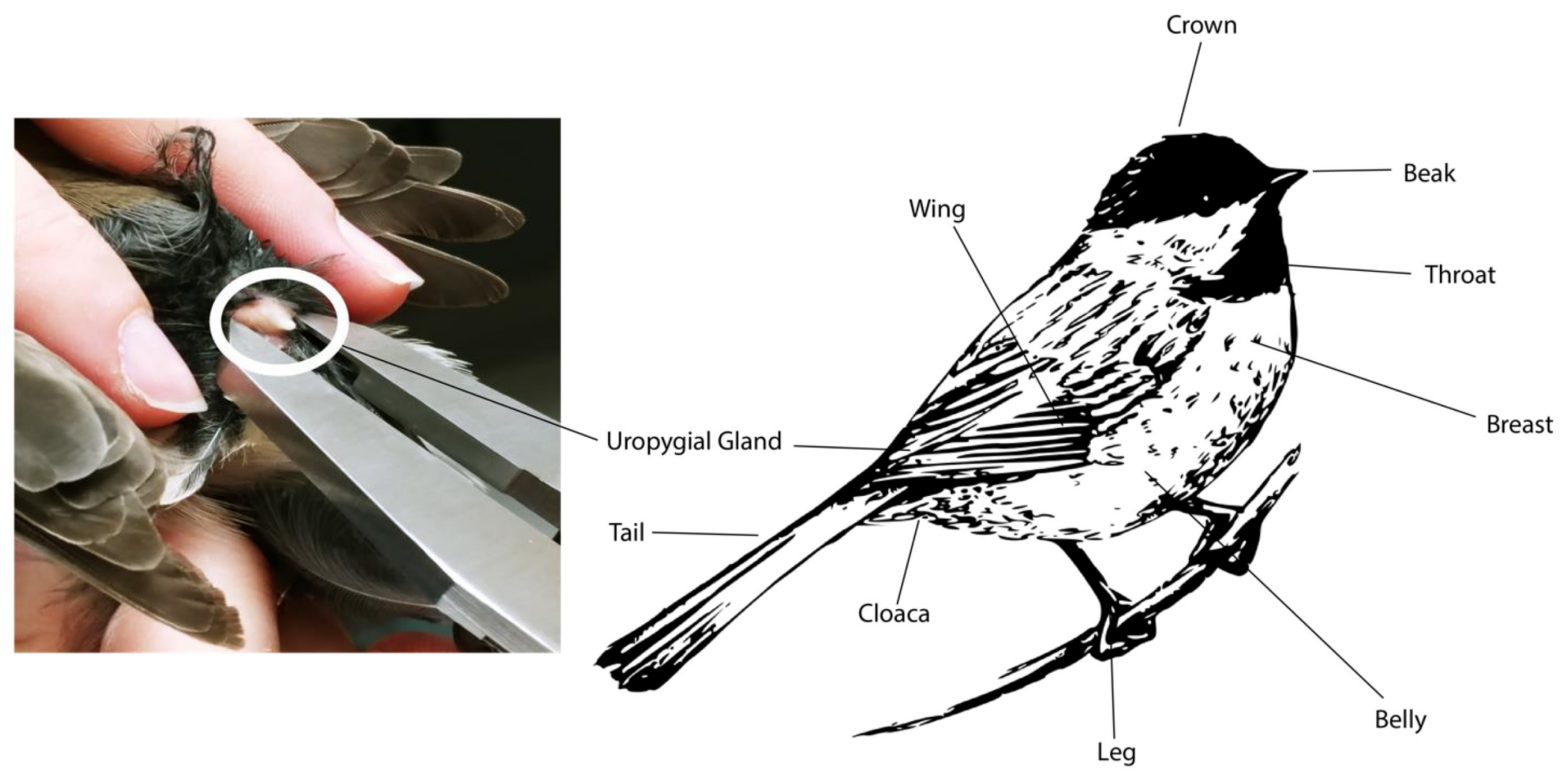
(Left) A white circle indicates the uropygial (preen) gland on a chickadee. (Right) An anatomical drawing of a chickadee (illustration obtained from Stockio.com) indicating the important anatomical locations of the songbird, including the uropygial gland and the cloaca.

We collected preen oil from a total of 12 individual birds, from 12 passerine species, spanning 10 genera, 8 families, and 1 order (Table 3). All these birds were captured in mist nets at bird feeders in Lehigh County, Pennsylvania, USA (U.S.G.S Federal Banding Permit 24256 and PA Game Commission Banding Permit 49864). We cleaned the uropygial gland (Figure 1) of each bird and temporarily cleared away any nearby feathers by wiping the area with a cotton swab that had been saturated with 100% ethanol. We then used forceps that were ethanol‐wiped before and after use to gently squeeze the gland. Once preen oil was secreted from the uropygial gland (Figure 1), we used a capillary tube to collect a small oil sample (~1–2 mg). After collection, the bottom of the capillary tube was placed in a 1.5 mL microcentrifuge tube and stored in a −80°C freezer prior to microbial DNA extraction.

**TABLE 3:** Avian taxa from which we obtained preen oil samples.

Order	Family	Genus	Species	Common name
Passeriformes	Corvidae	Cyanocitta	*Cyanocitta cristata*	Blue Jay
Passeriformes	Paridae	Poecile	*Poecile carolinensis*	Carolina chickadee
Passeriformes	Paridae	Poecile	*Poecile atricapillus*	Black‐capped chickadee
Passeriformes	Turdidae	Turdus	*Turdus migratorius*	American robin
Passeriformes	Sittidae	Sitta	*Sitta carolinensis*	White‐breasted nuthatch
Passeriformes	Fringillidae	Carduelis	*Carduelis tristis*	American goldfinch
Passeriformes	Fringillidae	Carpodacus	*Carpodacus mexicanus*	House finch
Passeriformes	Cardinalidae	Cardinalis	*Cardinalis cardinalis*	Northern cardinal
Passeriformes	Passerellidae	Zonotrichia	*Zonotrichia albicollis*	White‐throated sparrow
Passeriformes	Passerellidae	Melospiza	*Melospiza melodia*	Song sparrow
Passeriformes	Icteridae	Agelaius	*Agelaius phoeniceus*	Red‐winged blackbird
Passeriformes	Icteridae	Molothrus	*Molothrus ater*	Brown‐headed cowbird

All capture and sampling procedures were approved either by Lehigh University's Institutional Animal Care and Use Committee (Protocol #237) or by DeSales University's Institutional Animal Care and Use Committee (Protocol #1).

### 2.2 DNA extractions

To consistently extract microbial DNA from our avian fecal and preen oil samples, we revised the original Applied Biosystems™ PrepMan™ Ultra Sample Preparation Reagent method for extractions from bacterial samples, adding a bead beating step. For our fecal samples, we first removed approximately 0.15 g of feces from each sample, taking care to minimize collection of the white uric acid. After pouring any ethanol off the fecal sample, we placed the sample in a weigh boat in a fume hood for about 1 min to allow the evaporation of excess ethanol. We then transferred the fecal sample into a 2 mL Fisherbrand™ Free‐Standing Microcentrifuge Tube with Screw Cap that had been filled with approximately 100 μL of 460 nm acid‐washed glass beads (Sigma‐Aldrich, Inc., St. Louis, MO, USA) and 200 μL of PrepMan™ Ultra Sample Preparation Reagent (Thermo Fisher Scientific, Waltham, MA, USA). For preen oil samples, we placed the entire capillary tube tip (containing the preen oil sample) into a 2 mL microcentrifuge tube containing the same volumes of acid‐washed glass beads and PrepMan™ Ultra Sample Preparation Reagent as used with the fecal samples. Both types of sample mixtures were homogenized using a Mini‐Beadbeater‐8 (BioSpec Products Cat. No. 693, Bartlesville, OK, USA) for 1 min at 3450 oscillations/min (homogenize setting), and then placed in a water bath for 10 min at 100°C. For preen oil samples, we removed the capillary tube tip after the water bath step, at which point the oil sample was no longer observable in the tip of the capillary tube. For both sample types, we then centrifuged samples at 10,956 *g* for 2 min and pipetted off the supernatant. This supernatant was then used as the DNA extract for PCR. The volume of the final DNA extract samples was ~75 μL, depending on how much could be pipetted off without collecting the acid‐washed beads. To create a negative control, we followed this protocol using 100 μL of water instead of a fecal sample. For a positive control, a colony of *Escherichia coli* was prepared and used for extraction and PCR amplification.

### 2.3 PCR amplification of microbial 16S rRNA

To assess the success of our microbial DNA extraction method, we performed PCR amplification of hypervariable regions of the 16S rRNA gene (V3–V4 regions) using our DNA extracts from both fecal and preen oil samples (Bodawatta, Freiberga, et al., 2021; Bukin et al., 2019; Hird et al., 2015; Janda & Abbott, 2007; Srinivasan et al., 2015). For each of the fecal extractions, the non‐diluted sample was amplified by PCR and run on an agarose gel; however, if the sample did not show even a faint band, the sample was diluted, as this has shown to be beneficial for samples with PCR inhibitors (Eriksson et al., 2017). For eight of our 15 microbial DNA extractions from fecal samples, we mixed 1 μL of the concentrated fecal DNA sample with 99 μL of nuclease‐free water. Due to the small volume of collected preen oil samples (~1–2 mg), we did not dilute the preen oil DNA extracts.

We used a total PCR volume of 20 μL, containing master mix, GC enhancer, forward and reverse 16S rRNA primers, DNA, and water. Specifically, each reaction included 10 μL Platinum™ II Hot‐Start Green PCR Master Mix (2×) from Invitrogen (Waltham, MA, USA), 4 μL of the Platinum GC Enhancer included with this master mix, 2 μL of nuclease‐free H_2_O, and 2 μL of our DNA extraction. To amplify the V3–V4 region of the 16S rRNA gene, we also included 1 μL of both 25 μM 341F (CCTACGGGNGGCWGCAG) (Klindworth et al., 2013) and 806R (GACTCHVGGGTATCT‐AATCC) (Caporaso et al., 2011) 16S rRNA primers at 25 μM stock concentration, resulting in a final concentration of 1.25 μM for each primer in each reaction.

Optimized PCR conditions included an initial denaturation step at 95°C for 2 min, followed by 30 cycles of denaturation at 95°C for 30 s, annealing at 55°C for 30 s, extension at 68°C for 1 min, and a final elongation at 68°C for 2 min. To confirm successful PCR amplification, we ran each sample on a 2% agarose gel at 140 V for 50 min and verified the presence of bands visually. The expected product size was ~430 bp. For any extractions that did not show a band on the first agarose gel, we ran a new PCR reaction using the original DNA extract. We therefore did not extract DNA multiple times from any of our samples.

### 2.4 Sequencing library preparation

Once we established the successful amplification of the V3–V4 regions of the 16S rRNA gene from our DNA extracts using an agarose gel (see above), we further confirmed that our method successfully extracted microbial DNA by sequencing these regions from a small subset of our samples. Specifically, we sent seven PCR samples from two chickadee species (*Poecile atricapillus* and *Poecile carolinensis*) plus the 16S rRNA primers described above to Rush Genomics and Microbiome Core Facility (Chicago, IL, USA) for sequencing. Library preparation and sequencing of the seven samples were completed by the sequencing facility using the CS1 (ACACTGACGACATGGTTCTACA **CCTACGGGNGGCWGCAG)** and CS2 (TACGGT‐AGCAGAGACTTGGTCT **GACTCHVGGGTATCTAATCC)** linkers, indicated by underlining, on the 341F and 806R 16S primers, indicated in bold. The sequencing facility performed Fluidigm amplicon library preparation to ready the samples for next‐generation sequencing. The samples were normalized, pooled, and sequenced on Illumina MiSeq using paired‐end 300 bp reads. With each run, the sequencing facility ran a negative, no template control, and positive library controls alongside our samples to control for contaminants at different stages of the sequencing.

### 2.5 Sequencing data analysis

We followed a previously published pipeline (Lee, 2019) and used the R package ‘phyloseq’ (McMurdie & Holmes, 2013) for the statistical analysis of our microbial sequence data. We used the program FastQC to check the initial quality of the sequenced samples and to trim primer sequences. To keep a minimum Phred score of 25, we merged the forward sample at 275 bp and cut the reverse at 225 bp. Following the dada2 approach, bimeras were removed and taxonomy was assigned using the SILVA v138 reference taxonomy dataset (Quast et al., 2013). ASVs (amplicon sequence variants) were then assigned by dada2 and used for analysis. The package decontam was used to remove the sequences that appear more often in the negative controls than in the samples (Davis et al., 2018). However, due to the small number of samples and the single negative used for sequencing, this step may not be as stringent or accurate as with a larger dataset. Our R script is available on GitHub (https://github.com/rusty‐russ/Russell‐et‐al.‐Methods‐Paper).

## 3 RESULTS

**FIGURE 2:**
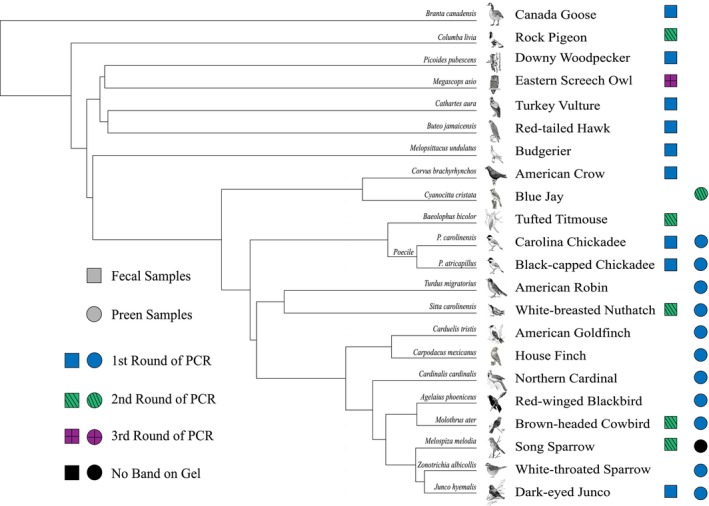
Phylogenetic relationships of the avian taxa from which we obtained fecal and preen samples. The phylogenetic tree was made using Vertlife.org. The circle indicates a preen oil sample was collected from that species and the square indicates a fecal sample was collected from that species. The number of PCR rounds run on the original extracted DNA to successfully see a band on a 2% agarose gel is indicated by the color and pattern within each circle or square. The V3–V4 region of the microbial 16S rRNA gene successfully amplified from all but one of the DNA extracts we generated using our method. See Figures S1–S5 for gel images.

Our extraction method was successful in extracting microbial DNA from both fecal and preen oil samples collected across a diverse range of avian taxa (e.g., songbirds, waterfowl, scavengers, and birds of prey), covering a broad range of feeding guilds (Tables 2 and 3; Figure 2). The success of our extraction method was evident by our consistent ability to PCR amplify the V3–V4 region of the microbial 16S rRNA gene, indicated by the presence of bands on agarose gels representing PCR products of the expected length (Figure 2; Figures S1–S5). Microbial DNA was only extracted once from each preen and fecal sample; however, multiple PCRs were occasionally necessary (Figure 2; Figures S1–S5) to visibly see bands on a 2% agarose gel indicating the presence of the microbial DNA. Out of 25 fecal collections from 15 species and 12 preen oil collections from 12 species, only the extraction from the single song sparrow preen oil sample did not produce a band on an agarose gel (Figure 2).

**FIGURE 3:**
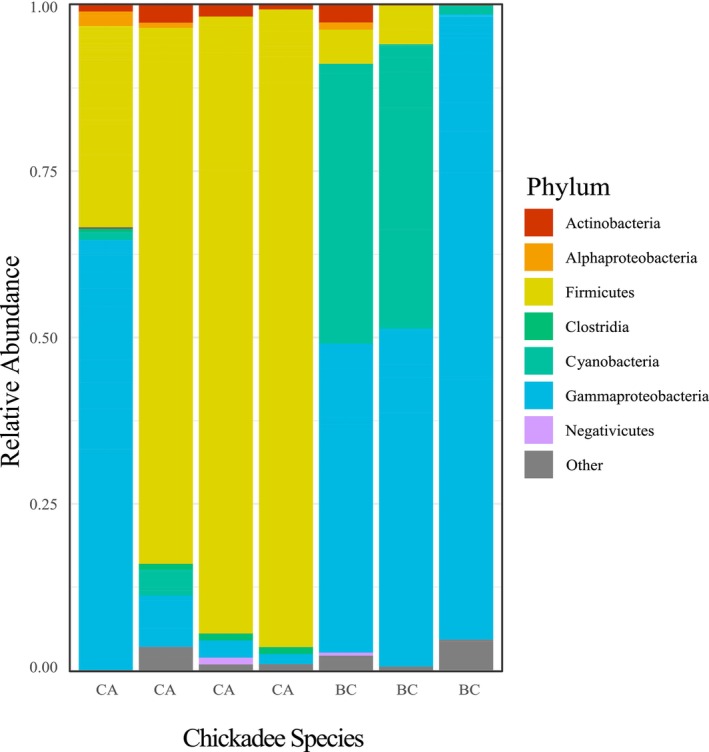
Relative abundance of several bacterial classes present in the microbial DNA extracted from three black‐capped (BC; *Poecile atricapillus*) and four Carolina chickadee (CA; *Poecile carolinensis*) fecal samples. See text for an explanation of how the microbial species present were identified using sequence data.

We measured the DNA concentration present in all samples by using the Qubit Flex Fluorometer (Table 4). The mean microbial DNA concentration from fecal collections equaled 3.142 ng/μL (±5.245 ng/μL SD) and the mean microbial DNA concentration from preen oil collections equaled 0.2598 ng/μL (±0.331 ng/μL SD). The DNA concentrations we obtained from fecal extractions are similar, if not higher, than concentrations obtained using many of the mainstream methodologies (Edwards et al., 2023; Eriksson et al., 2017). Additionally, the concentration values for our preen sample extractions are close to, if not higher, than some of the known published concentrations obtained using common kits (Table 4; Grieves, Gloor, Kelly, et al., 2021). Our successful amplification of the targeted 16S rRNA microbial gene regions was further confirmed by sequencing a subset of our PCR products. Specifically, we sequenced 16S rRNA V3–V4 regions in microbial DNA extractions done with fecal material collected from black‐capped and Carolina chickadees (*n* = 7). We obtained consistently high‐quality reads with a mean of 108,000 reads per sample. We obtained an initial count of 417 ASVs. Five of seven samples had at least 90% reads retained, while the remaining two had approximately 85% reads retained. Additionally, after trimming and merging and removing contaminants, there remained a count of 244 ASVs. Although we removed a larger than normal number of contaminants, as we note above, it is likely that the decontam method we used would be more accurate in its contaminant analysis with a larger sample size and the inclusion of more than one negative control. Given that our goal for sequencing this small number of samples was simply to show that a full walkthrough of standard microbiome methods would be successful for microbial DNA extractions generated using this methodology, we believe we achieved our goal. Subsequent bioinformatic analysis of the sequencing results revealed that our extraction method was successful in extracting both Gram‐positive and Gram‐negative bacteria (Table 5). Further, our results identifying the bacterial classes present in each extraction, as well as the relative abundance of different bacterial classes, suggest that the gut microbiome can vary across different chickadee individuals (Figure 3).

**TABLE 4:** Concentration of microbial DNA from preen oil extractions (left) and concentration of microbial DNA from avian fecal extractions (right). Performed using a high sensitivity buffer on the Qubit Flex Fluorometer.

Preen sample ID	DNA concentration (ng/μL)	Fecal sample ID	DNA concentration (ng/μL)
White‐throated sparrow	<0.05	Brown‐headed cowbird	0.471
House finch	<0.05	Downy woodpecker	3.42
American robin	<0.05	Tufted titmouse: Individual 1	2.31
Red‐winged blackbird	0.143	Tufted titmouse: Individual 2	1.75
American goldfinch	0.0642	Canada goose: Individual 1	1.56
Song sparrow	1.07	Canada goose: Individual 2	0.695
Brown‐headed cowbird	0.145	Dark‐eyed junco: Individual 1	0.433
Northern cardinal	0.488	Dark‐eyed junco: Individual 2	0.496
Blue Jay	0.611	Carolina chickadee	0.685
Carolina chickadee	0.13	White‐breasted nuthatch	1.19
Black‐capped chickadee	0.143	Black‐capped chickadee	0.611
White‐breasted nuthatch	0.233	Song sparrow	0.754
		Red‐tailed hawk	4.33
		Eastern screech owl	3.24
		Turkey vulture	13.7
		Budgerigar: Individual 1	21.6
		Budgerigar: Individual 2	0.51
		Rock pigeon: Individual 1	2.18
		Rock pigeon: Individual 2	2.15
		American crow	0.757

**TABLE 5:** Gram‐positive and Gram‐negative categorization of the bacterial taxa detected from the amplification and sequencing of the 16S rRNA gene from seven microbial DNA samples extracted using our method.

Gram‐positive	Gram‐negative
Acidimicrobiia	Acidobacteriae
Alphaproteobacteria	Alphaproteobacteria
Actinobacteria	Bacteroidota
Firmicutes	Bdellovibrionota
	Chloroflexi
	Cyanobacteriia
	Firmicutes
	Fusobacteriota
	Gemmatimonadota
	Planctomycetota
	Pseudomonadota
	Verrucomicrobia

## 4 DISCUSSION

Our modified microbial DNA extraction method is effective for avian fecal samples (Figure 2, Table 4), which have been traditionally challenging materials for use in DNA extractions (Eriksson et al., 2017; Hou et al., 2021; Torok et al., 2008). Our method is also effective for preen gland oil samples (Figure 2, Table 4), which likely harbor lower bacterial abundance due to antimicrobial characteristics of preen oil (Martín‐Vivaldi et al., 2009). Both sample types were collected across a broad range of avian species. Further, our method successfully extracted DNA from both Gram‐positive and Gram‐negative bacteria (Table 5). Other studies have found both Gram positive and negative bacteria in avian fecal samples, supporting the efficacy of this method (Bodawatta, Puzejova, et al., 2020; Davidson et al., 2020; Kohl, 2012; Videvall et al., 2018; West, DeLaunay, et al., 2022). The ease and relative cost effectiveness of our method (Table 1) makes it particularly advantageous. We hope that our method will facilitate advances in our understanding of microbiomes from multiple reservoirs across a wide range of avian species.

While an organism's host microbiome is a crucial determinant of its overall health, the full extent of the coevolutionary relationships between a host and its symbiotic microbes is not known (Bodawatta, Hird, et al., 2021; Broom & Kogut, 2018; Loftus et al., 2021; Molinero et al., 2022; Shreiner et al., 2015). Recent discoveries have shown hosts rely on microbes for health and protection against viruses and pathogens, providing enzymes that break down molecules (Bahrndorff et al., 2016; Broom & Kogut, 2018; Kau et al., 2011; Loftus et al., 2021; Shreiner et al., 2015). Additionally, hosts rely on microbes for digestion, cognitive function, growth, development, and more (Bodawatta, Hird, et al., 2021; Davidson et al., 2020; Grond et al., 2018; Ikeda‐Ohtsubo et al., 2018; Kohl, 2012; Sommer & Bäckhed, 2013). Gut microbiomes are particularly important in many taxa (Hasan & Yang, 2019; Loo et al., 2019; Molinero et al., 2022; Rosshart et al., 2017). To sample the gut microbiome of birds, cloacal swabs have been shown to be nonrepresentative of microbial diversity, only accounting for one component of the digestive tract, whereas fecal samples contain inputs from the whole gastrointestinal tract and are thus more accurate representations of the entire gut microbiome (Eriksson et al., 2017; Videvall et al., 2018). However, existing DNA extraction methods have not been consistently successful when working with avian fecal samples (Eriksson et al., 2017; Vo & Jedlicka, 2014). Birds and reptiles combine their uric waste with their fecal waste, thus creating added difficulty in extracting DNA from fecal samples (Pearson et al., 2015). Our method is not only reliable with such samples but is considerably cheaper than most commercial kits used to extract avian microbiome DNA (Table 1). The low cost and high reliability of our method will improve the feasibility and accessibility of research on avian host microbiomes.

As microbiome studies have increased in popularity over the last decade, the range of host reservoirs scientists are studying has expanded as well. Beyond the abundant and diverse gut microbiome, a potentially functionally important reservoir in avian species is the microbiome that exists within the uropygial or preen gland (Grieves, Gloor, Bernards, et al., 2021). The preen gland and the oils produced there are known to affect communication, species recognition, mate choice, and feather maintenance (Grieves, Gloor, Kelly, et al., 2021; Huynh & Rice, 2019; Whittaker et al., 2010). The chemical composition of preen oil is at least partially dependent on the microbes present in this gland (Caspers et al., 2022; Huynh & Rice, 2019, 2021; Whittaker et al., 2019). The bacteria present in the uropygial microbiome are thought to be less abundant than those within the gut microbiome; yet they are predicted to play direct roles in the odor of avian hosts as well as potentially in feather and body health (Jacob et al., 2014; Møller & Laursen, 2019; Whittaker et al., 2013, 2019). Some preliminary studies in this field of research have observed that disease may not be directly linked to differences in preen oil microbiomes (Videvall et al., 2021). Additionally, differences in microbial communities may correspond with population differences, and in some species, sex differences have been observed (Rodríguez‐Ruano et al., 2018). The consistent success of our method in extracting microbial DNA from avian preen oil samples will facilitate new research on this currently understudied microbiome reservoir.

Because we have established the consistent success of our method in extracting microbial DNA from two avian sample types that are quite chemically different, it is likely that our method will be useful across additional avian microbiome reservoirs. Current variation in the approaches used for microbial DNA extraction and subsequent amplification means there is also sometimes variation in the bacteria that are detected (Eriksson et al., 2017; Gerasimidis et al., 2016). Using the same extraction methodology across samples from different reservoirs will allow more consistent and confident comparisons of microbiomes from different reservoirs for future research efforts. With increased interest in the relationship between hosts and their bacterial community composition and abundance across different microbiome reservoirs, the accurate identification of microbial taxa is critical. With an enhanced potential to identify and quantify the symbiotic microbes, the investigation of their impact on host health and conservation can involve deeper questions. Widespread adoption of our method would therefore increase the validity of future cross‐study and cross‐reservoir comparisons.

To conclude, we have developed and tested a highly efficient microbial extraction method and verified its efficacy across multiple avian species. Our method was successful in extracting microbial DNA on the first attempt from 25 of 25 fecal samples collected from 15 avian species and from 11 of 12 preen oil samples collected from 12 avian species (Figure 2, Tables 2 and 3; Figures S1–S5). It is also relatively inexpensive compared with commercial fecal DNA extraction kits (Table 1). We are confident that our method will be effective and efficient across many additional avian species globally. Future work should explore the effectiveness of this method in extracting microbial DNA from reservoirs in other taxa, including reptile fecal samples as well.

## AUTHOR CONTRIBUTIONS

**Austin C. Russell:** Conceptualization (lead); data curation (lead); formal analysis (lead); investigation (lead); methodology (equal); visualization (lead); writing – original draft (equal); writing – review and editing (lead). **Margaret A. Kenna:** Conceptualization (supporting); data curation (supporting); investigation (equal); methodology (equal); resources (supporting); writing – review and editing (supporting). **Alex Van Huynh:** Conceptualization (supporting); data curation (equal); investigation (supporting); methodology (supporting); resources (supporting); writing – review and editing (supporting). **Amber M. Rice:** Conceptualization (supporting); data curation (supporting); formal analysis (supporting); funding acquisition (lead); investigation (supporting); methodology (supporting); project administration (lead); resources (lead); supervision (lead); writing – original draft (supporting); writing – review and editing (supporting).

## CONFLICT OF INTEREST STATEMENT

The authors of this paper have no conflicts of interest.

## BENEFIT‐SHARING STATEMENT

Benefits Generated: Benefits from this research accrue from the sharing of our data and results on public databases as described above.

## Supporting information


Figures S1–S5

## Data Availability

*Genetic data*: Raw sequence reads are deposited in the NCBI Nucleotide Database (PRJNA1015692). *Sample metadata*: Metadata are also stored in NCBI Nucleotide Database (PRJNA1015692).
