# Supplementary material for: Microbial DNA extraction method for avian feces and preen oil from diverse species
Source: Ecol Evol. 2024 Sep 2;14(9):e70220. doi: 10.1002/ece3.70220 (PMC11368492; doi:10.1002/ece3.70220)
Supplement: Supplementary file 1 — Figures S1–S5 [file ECE3-14-e70220-s001.docx]

**Supplemental Figures:**


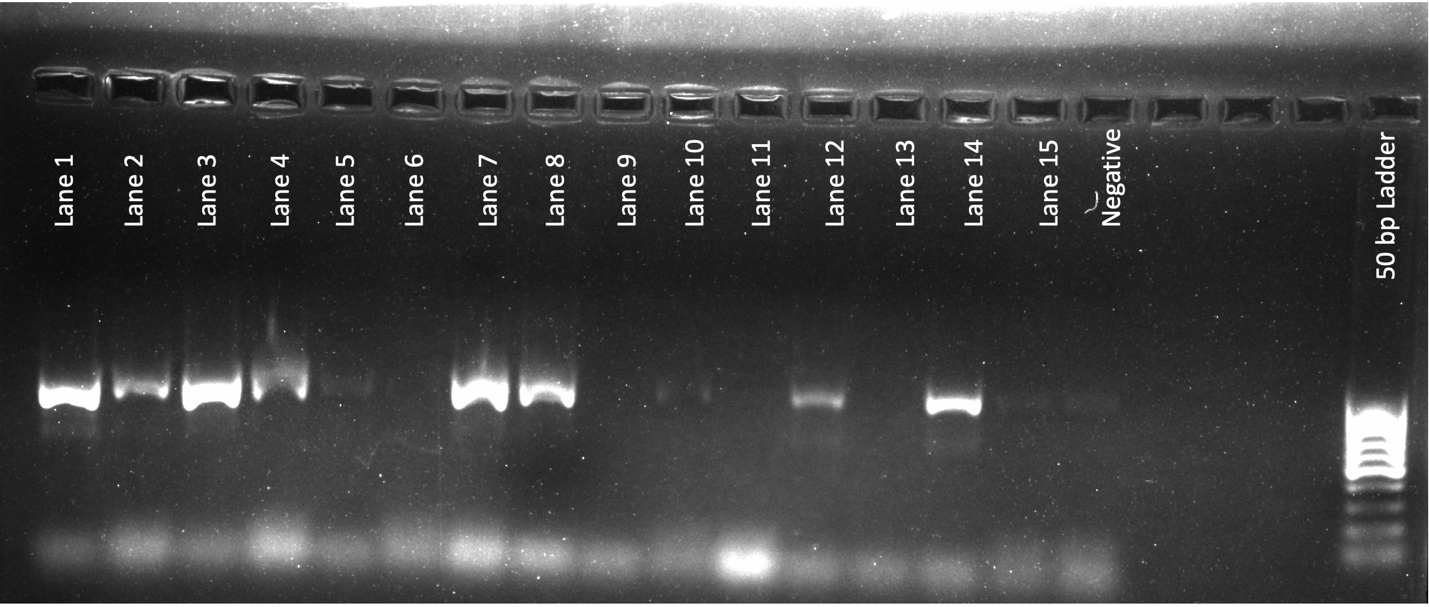


**Figure S1**: Gel 1, containing bands representing the V3-V4 region of the 16S rRNA gene amplified by PCR using DNA extracts from fecal samples. The common species names for the samples listed by lane number from left to right are (1) Canada goose, (2) Rock pigeon 1:100 dilution, (3) Turkey vulture, (4) Red-tailed hawk, (5) Downy woodpecker, (6) eastern screech owl, (7) Budgerigar 1:100 dilution, (8) American crow, (9) Tufted titmouse 1:100 dilution, (10) Carolina chickadee 1:100 dilution, (11) Black-capped chickadee 1:100 dilution, (12) White-breasted nuthatch 1:100 dilution, (13) Brown-headed cowbird 1:100 dilution, (14) Dark-eyed junco 1:100 dilution, (15) Song sparrow, and (16) water negative. On the right is a 50 bp ladder.


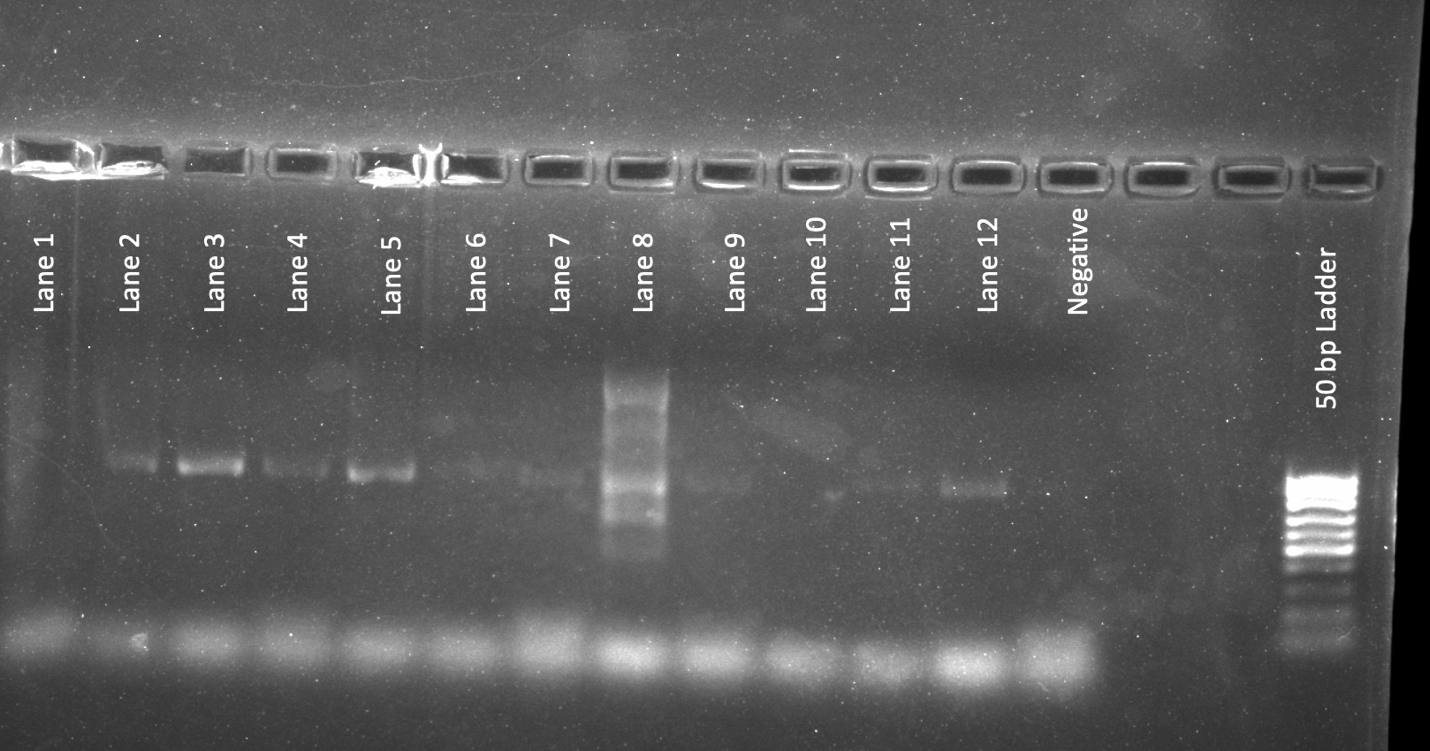


**Figure S2**: Gel 2, containing bands representing the V3-V4 region of the 16S rRNA gene amplified by PCR using DNA extracts from preen oil samples. The common species names for the samples listed by lane number from left to right are (1) Blue Jay, (2) Black-capped chickadee, (3) Carolina chickadee, (4) American Robin, (5) White-breasted nuthatch, (6) American goldfinch, (7) House finch, (8) Northern cardinal, (9) White-throated sparrow, (10) Song sparrow, (11) Red-winged blackbird, (12) Brown-headed cowbird, (13) water negative. On the right is a 50 bp ladder.

**
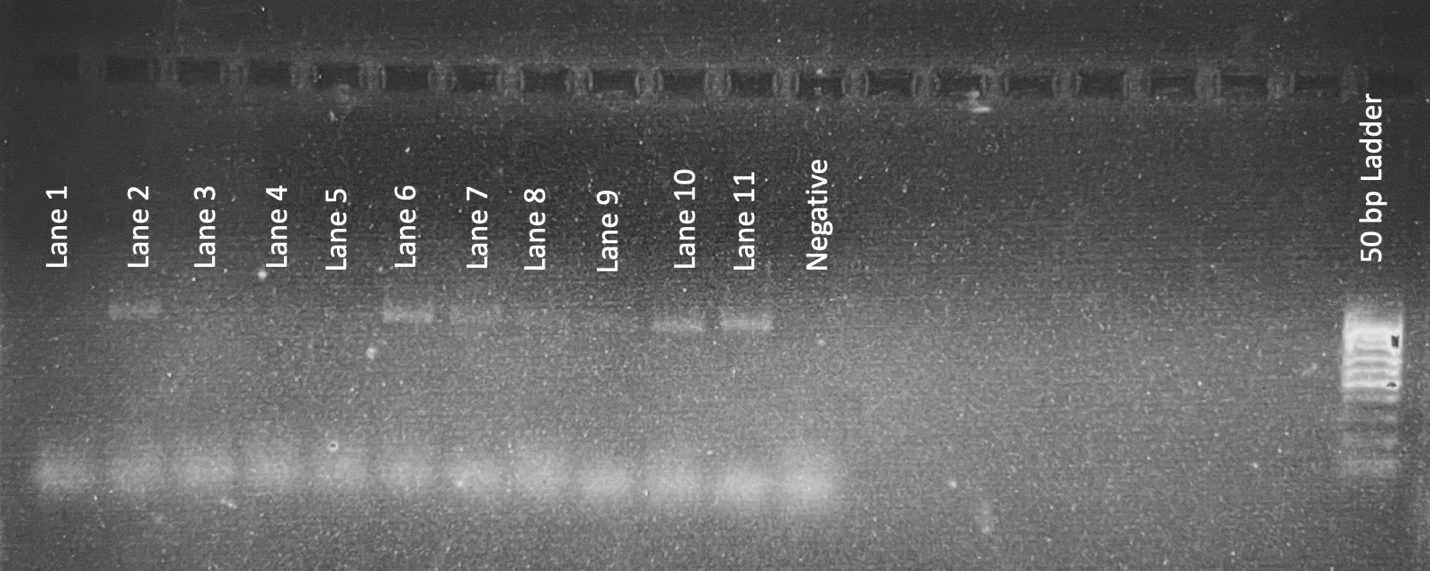
**

**Figure S3:** Gel 3, containing bands representing the V3-V4 region of the 16S rRNA gene amplified by PCR using DNA extracts from additional fecal samples. The common species names for the samples listed by lane number from left to right are (1) Rock Pigeon, (2) Rock Pigeon 1:100 dilution, (3) Eastern Screech Owl, (4) Eastern Screech Owl 1:100 dilution, (5) Tufted Titmouse, (6) Tufted Titmouse 1:100 dilution, (7) White breasted nuthatch, (8) White breasted nuthatch 1:100 dilution, (9) Brown-headed cowbird, (10) Brown-headed cowbird 1:100 dilution, (11) *E. coli* positive control, (12) water negative control. On the right is a 50 bp ladder.


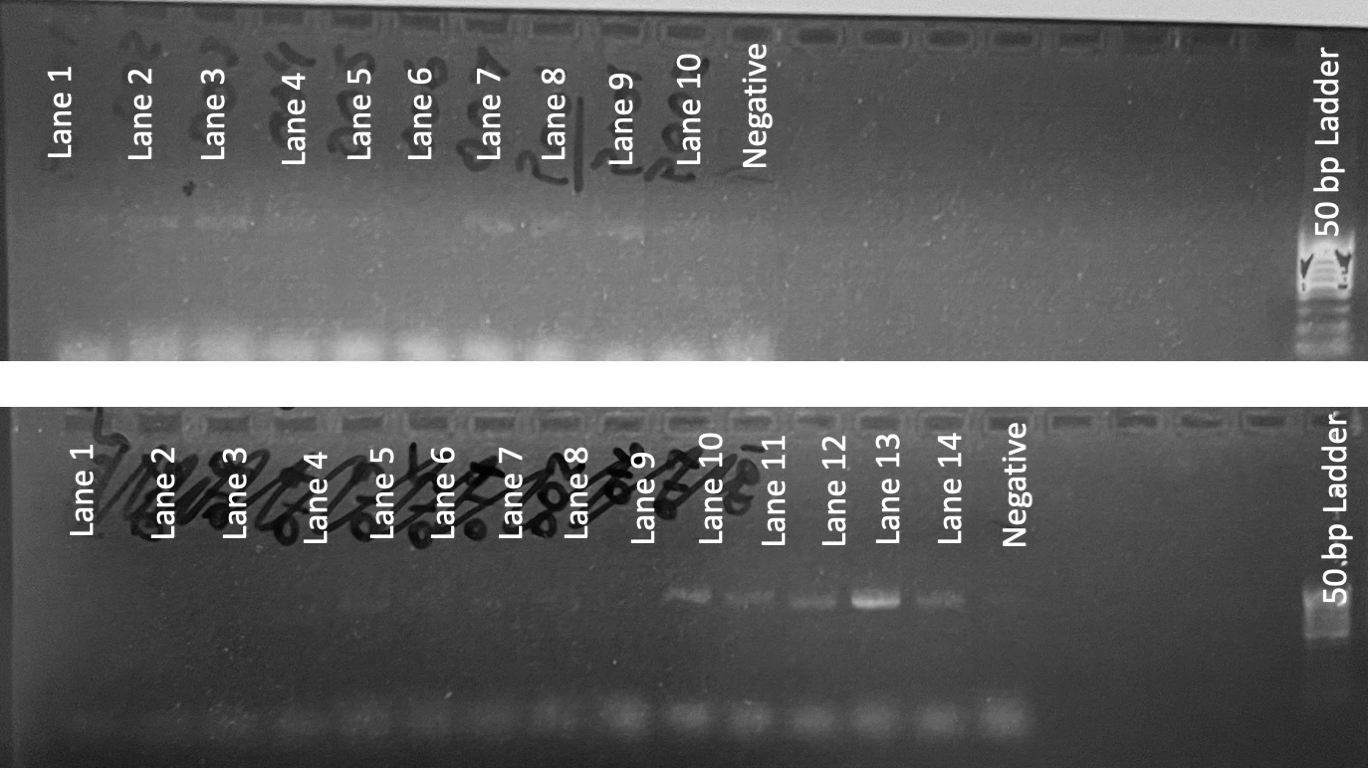


**Figure S4:** Gel 4, containing bands representing the V3-V4 region of the 16S rRNA gene amplified by PCR using DNA extracts from additional fecal and preen oil samples. The common species names for the samples listed by lane number from left to right in the image are (1) Brown-headed cowbird fecal, (2) Downy woodpecker fecal, (3) Tufted titmouse fecal, (4) Tufted titmouse fecal, (5) Carolina chickadee fecal, (6) Dark-eyed junco fecal, (7) Dark-eyed junco fecal, (8) Carolina chickadee fecal, (9) Eastern screech owl fecal, (10) Black-capped chickadee preen, (11) Carolina chickadee preen, (12) White-breasted nuthatch preen, (13) Black-capped chickadee 1:100 dilution fecal, (14) Black-capped chickadee preen, and (15) water negative control. On the far right is a 50 bp ladder.

**
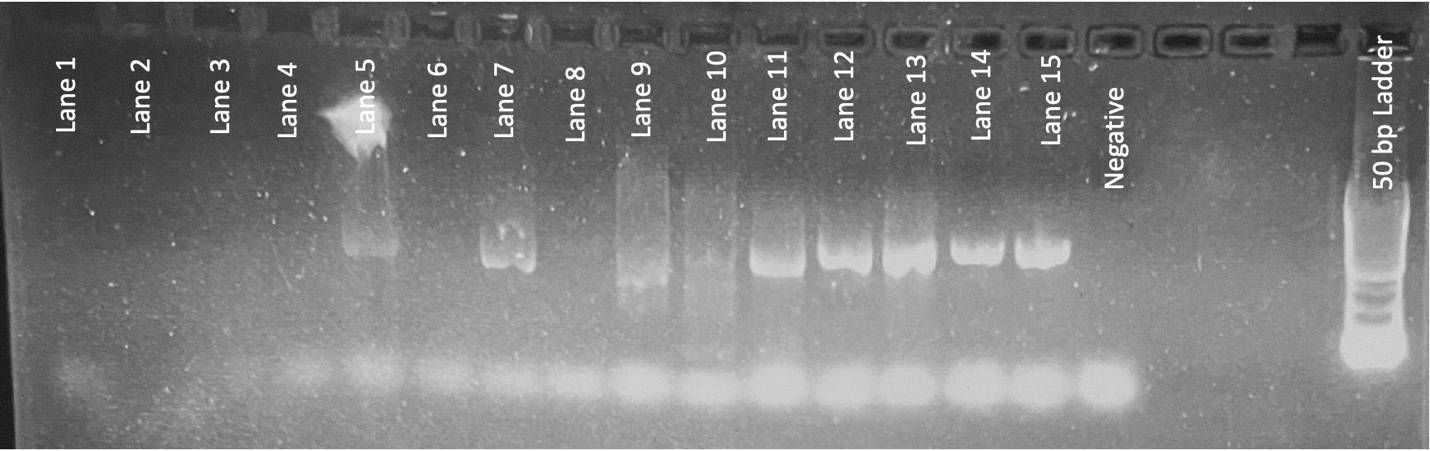
**

**Figure S5:** Gel 5, containing bands representing the V3-V4 region of the 16S rRNA gene amplified by PCR using DNA extracts from additional fecal and preen oil samples. The common species names for the samples listed by lane number from left to right are (1) Carolina chickadee fecal, (2) Dark-eyed junco fecal, (3) Song sparrow fecal, (4) Song sparrow fecal, (5) Red-tailed hawk fecal, (6) Eastern screech owl fecal, (7) Turkey vulture fecal, (8) White-throated sparrow preen, (9) Northern cardinal preen, (10) Blue Jay preen, (11) Budgerigar fecal, (12) Budgerigar 1:100 dilution fecal, (13) Budgerigar fecal, (14) Budgerigar 1:100 dilution fecal, (15) *E. coli* positive control and (16) water negative control. On the far right is a 50 bp ladder.
